# In-vitro study on type I collagen synthesis in low-level laser therapy on the early ligament fibroblasts’ healing process

**DOI:** 10.1007/s10103-024-04151-7

**Published:** 2024-08-29

**Authors:** R. P. Cárdenas-Sandoval, L. D. Bernal-Bernal, S. Cabrera-Salazar, D. M. Gómez-Ramírez, L. M. González-Ballesteros, K. M. Hooker-Mendoza, L. N. Ospina-Piedrahíta, C. X. Hernández-Charry, G. Ardila-Rojas, A. M. Velásquez-Durán, J. D. Cucarián-Hurtado, A. O. Ondo-Méndez, J. Barbosa-Santibañez, L.L. Carvajal-Calderón, M. L. Navarrete-Jimenez

**Affiliations:** 1 Rehabilitation Science Research Group, School of Medicine and Health Sciences, Universidad del Rosario, Bogotá, Colombia; 2 Neuroscience and Mental Health Institute and Faculty of Rehabilitation Medicine, University of Alberta, Edmonton, Alberta Canada; 3 Clinical Research Group, School of Medicine and Health Sciences, Universidad del Rosario, Bogotá, Colombia; 4 Clinical Research Group, Hospital Universitario Mayor Méderi, Bogotá, Colombia; 5 Department of Microbiology, Faculty of Medicine, Universidad Nacional de Colombia, Bogotá, Colombia

**Keywords:** Low-level laser therapy (LLLT), Anterior cruciate ligament, Fibroblasts, Cell viability, Cell proliferation, Collagen type I, Vimentin

## Abstract

**Background:**

Low-level Laser Therapy (LLLT) has demonstrated its potential in promoting fiber matrix maturation, collagen synthesis, and fibroblast proliferation, contributing to tissue regeneration. Our study aimed to investigate the impact of LLLT on collagen type I synthesis, cell proliferation, and viability in human ligament fibroblasts derived from the Anterior Cruciate Ligament (ACL).

**Methods:**

Tissue samples were obtained from individuals undergoing arthroscopic ACL reconstruction surgery. Primary human fibroblasts were isolated, and immunohistochemical assays confirmed their characteristics. LLLT at 850 nm was administered in three groups: Low dose (1.0 J/cm²), High dose (5.0 J/cm²), and Control (0.0 J/cm²). Cell viability was calculated using a membrane integrity assay, proliferation was determined by automated counting, and collagen type I concentration in cell culture was measured using an immunoassay.

**Results:**

Fibroblasts showed decreased viability after low and high doses of LLLT, increased proliferation at the low dose, and increased collagen synthesis at the high dose on day 10 for both sexes after treatment.

**Conclusion:**

Our study demonstrated that LLLT may improve the early ligament healing process by increasing cell proliferation at the low dose and enhancing collagen type I synthesis at the high dose in human ligament fibroblasts.

**Supplementary Information:**

The online version contains supplementary material available at10.1007/s10103-024-04151-7.

## Introduction

The extracellular matrix of connective tissues represents a combination of proteins that define structural integrity and various physiological functions [1]. Collagen is the most important because it contributes to the stability of organs and tissues to maintain their structure and integrity. Ligaments are connective tissues composed primarily of fibrillary collagen and extracellular matrix (ECM) [2]. Ligaments connect bone to bone and contribute to the structural form, the resistance of tensile loads, and joint stability. The main cellular components of the ligaments are the fibroblast, which can divide, migrate through ECM, and secrete collagen fibers [3].

The functional integrity of ligaments can also be affected by alterations in the nature and organization of collagen. It is caused by external factors such as trauma, postoperative, impaired functions, or adhesion in the wound healing process [4]. Tissue repair is a dynamic process comprising different processes, including inflammation, cellular proliferation, and synthesis of extracellular matrix elements, such as collagen, reticular, and elastic fibers [4, 5]. Fibroblasts are the primary cells leading the ligament healing process. However, internal factors such as sex, age, level of physical activity, and collagen diseases, among others, can affect the repair process. In addition, abnormal collagen deposition can alter the functionality, possibly due to adhesions during healing [5–7].

Low-Level Laser Therapy (LLLT) has been shown to influence cellular components that play a crucial role in microenvironment healing. It has been observed that this therapy stimulates the proliferation of fibroblasts, macrophages, lymphocytes, endothelial cells, and keratinocytes, thereby promoting the healing process. LLLT also promotes the maturation of fiber components in the matrix and collagen synthesis, contributing to the regeneration of affected tissues. Several authors have reported the beneficial effects of LLLT in various contexts, such as periodontal and orthognathic surgeries, as well as in the treatment of tissues, including tendons, urethral fibroblasts, and keratinocytes, among others [8–10].

An in vitro study of gingival fibroblasts results in significant collagen type I gene expression. Cell proliferation was not significantly different between the control and the treatment groups on the first day post-irradiation. However, cell proliferation events began after 24 h and became more significant at 48 and 72 h post-irradiation [11].

However, studies have not demonstrated the effect of LLLT on the healing of the human intraarticular ligament by changes in fibroblast proliferation and collagen type I expression. Also, it cannot be proved that the LLLT is a safe treatment for ligament impairments by decreasing rehabilitation timelines. [5–10].

This research introduces novel LLLT parameters that may improve collagen type I synthesis and cell proliferation in human ligament fibroblasts derived from the Anterior Cruciate Ligament (ACL). Interesting differences were observed between the behavior of ligament fibroblasts from the female and male young adult samples. Our research offers insights into LLLT parameters that could open new avenues in regenerative medicine and tissue engineering.

## Materials and methods

This study protocol was approved by the Ethical Committee for Research in Life Sciences, Universidad del Rosario, Bogotá, Colombia (approval number: DVO005 1842-CV1322).

### Explant technique and cell culture

Tissue samples were obtained from a 36-year-old woman and a 28-year-old man who received knee arthroscopy surgery to replace their injured ACL at the hospital. Each patient provided a signed written statement of informed consent. A few sections of a ruptured anterior cruciate ligament were obtained during the ACL replacement procedure. Every section was gathered and stored in a sterile falcon tube (50 ml) and cultured in Dulbecco’s modified Eagle Medium (DMEM-F12) with 4.5 g/L glucose, L-Glutamine with sodium pyruvate, 500 mL (Lonza, CA, USA), supplemented with 1% antibiotics/antifungals, and 10% fetal bovine serum (Gibco, Carlsbad, CA, USA).

The Falcon tube containing the tissue sample was meticulously transported in a portable cooler with ice packs to maintain a low temperature of approximately 4 °C. This was done to ensure the preservation of the sample’s integrity. We strictly adhered to the biological safety protocol during transportation, ensuring the sample’s safety and preventing potential contamination. The sample was safely transported to the Biochemical Laboratory at the University.

We followed a previously reported protocol for isolating human ACL [12, 13]. The explanted tissue was washed thrice in a sterile glass petri dish with 30 ml of a solution made with Phosphate-Buffered Saline (PBS). The tissue was then cut into smaller pieces using sterile forceps and placed in a T25 cell culture flask. The flask was left to dry for approximately 3 h in aseptic conditions to promote adherence. Once the explants were completely adhered to the flask surface, we gently added DMEM/F12. The growth of fibroblasts from the explant surfaces was monitored for 48 and 72 h until day 15 when the cells grew over the flask and reached 80% of cell confluence [13].

During this time, the cell cultures were maintained in DMEM-F12 supplemented; they were incubated at 37 °C in a high atmosphere with 5% CO2. The overlying liquid culture media was changed every 48 h with fresh culture media.

### Ligament fibroblasts verification

One hundred twenty thousand cells were seeded in a 12-well cell culture plate at an approximate density of 60% to perform an immunohistochemistry assay conducted on primary isolated ACL human fibroblasts. A human breast cancer cell line (MCF-7) and a mouse fibroblast line (NIH/3T3) obtained from the Biochemistry laboratory’s cell bank of the University were used as negative and positive controls. Cells plated in a 12-well cell culture plate were grown for 48 h in DMEM-F12 medium before being fixed (4% PFA) and permeabilized (0.1% Triton). After a blocking step (BSA 1%), immunofluorescence labeling was performed using a mouse monoclonal anti-vimentin antibody at 1:50 (AlexaFluor^®^ Invitrogen) and a goat anti-mouse 488- secondary antibody 1:1000 (AlexaFluor^®^ Invitrogen). Nuclei were stained with Hoechst 33,342 (Invitrogen H3570). Fluorescence images were captured and analyzed using a Cytation3 imaging reader (Agilent BioTek) [10].

### Low-level laser therapy irradiation protocol

**Fig. 1:**
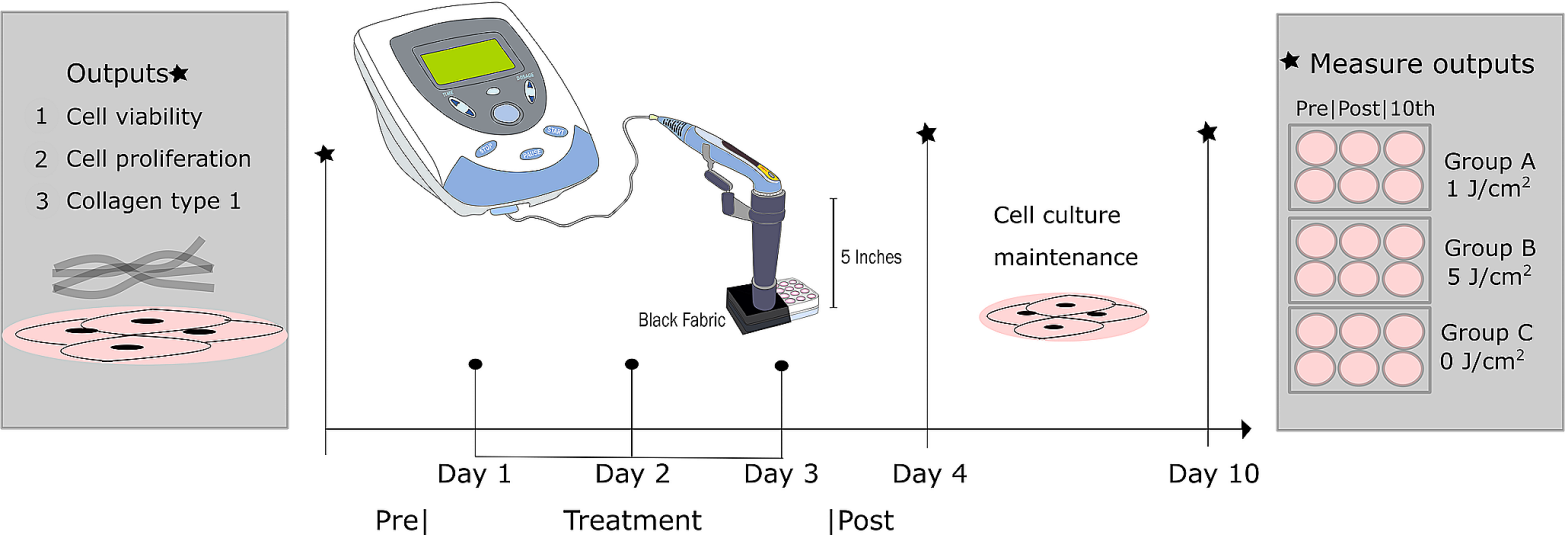
LLLT protocol irradiation every 24 h for three consecutive days

The Low-Level Laser Therapy (LLLT) was performed using a Chattanooga Intelect^®^ mobile therapeutic laser device with a wavelength of 850 nm (Table 1). We established three irradiation groups: Low dose (1.0 J/cm^2^), High dose (5.0 J/ cm^2^), and a control group (non-irradiated cells). Cells were plated in a 6-well plate and grown for 24 h in DMEM-F12 supplemented medium before irradiation. An external adaptive device was designed using polylactic acid thermoplastic and 3D printing, which included a collimator lens with a focal length of 5 inches to ensure uniform irradiation over the sample (Fig. 1). During irradiation, a black fabric was used to prevent the cells from being exposed to external light. All treated groups were irradiated every 24 h for three days. The control group was not irradiated but was exposed to the same irradiation area and environmental conditions.

**Table 1:** LLLT parameters of the treatment doses. J/cm²: Joules/centimeter; W/cm²: Watts/centimeter; min: minute

Treatment groups	Low Dose	High Dose	Control group
Wavelength (λ) (nm)	850	850	0
Energy density [E] (J/cm²)	1.0	5.0	0
Power [P] (W/cm²)	100	100	0
Cycle (%)	100	100	0
Time (min)	0.26	2.06	2.06

### Cell proliferation and viability testing

After the irradiation protocol, cells were trypsinized (0,05% trypsin/EDTA in PBS for 5 min) and counted with an automated image-based cell counter (Corning^®^ Cell Counter). The cell density of the first column of each six-well plate was measured on the first day of pre-treatment, the second column on the fourth day post-treatment, and the third column on the tenth day after treatment (Fig. 1). Cell viability was assessed by Trypan blue staining, following the protocol provided by The National Institute of Environmental Health Sciences (NIEHS) [14]. The obtained cell count gave us the total number of live cells, indicating the viability percentage in each treatment after laser irradiation.

### ELISA for the quantitative measurement of collagen type I

As described earlier, the protocols for human ACL isolation [15, 16] and irradiation were carried out as a preliminary step to performing the collagen synthesis assay. During the irradiation protocol, the medium was collected and stored in cryovials at -4ºC on day one before and on days 4 and 10 after treatment. These collections were performed at the same time each day.

Enzyme-linked immunosorbent assays (ELISA) are laboratory techniques that use plates to detect and measure the presence of a specific protein in a complex sample. In our study, we have employed the type of ELISA known as “sandwich.” This name is given because the measured antigen is sandwiched between two primary antibodies. This provides greater sensitivity and specificity as specific monoclonal antibodies are used for antigen capture and detection [17].

We utilized an ELISA kit to evaluate the in vitro quantitative determination of the Human Collagen Type I Alpha 1 (COL1α1) ELISA Kit (Elabscience, United States). The kit has a detection range of 0.31-20 ng/mL. Optical density (OD) was measured spectrophotometrically at a wavelength of 450 nm ± 2 nm using Absorbance Microplate-Reader AMR-100 ALL SHENG. The concentration of Human COL1α1 in the samples was calculated by comparing their OD values to the standard curve.

### Statistical analysis

All statistical analyses were processed using SPSS^®^ ver. 25.0 (IBM Inc., Armonk, New York, USA). Triplicate assays were conducted in each experiment to mitigate the potential influence of small sample sizes on result validity and ensure greater robustness in data interpretation. The data were presented as mean and standard deviation. Initially, a sample normality test was conducted, followed by a non-parametric analysis using the Kruskal-Wallis test to determine between-group differences. Additionally, changes in the means of the variables were expressed in terms of percentage differences. Finally, a p-value of 0.05 was used to test for statistical significance. The graphs were generated using GraphPad Prism 5.0 (GraphPad Software, Inc., San Diego, CA).

## Results

### Immunohistochemistry

**Fig. 2:**
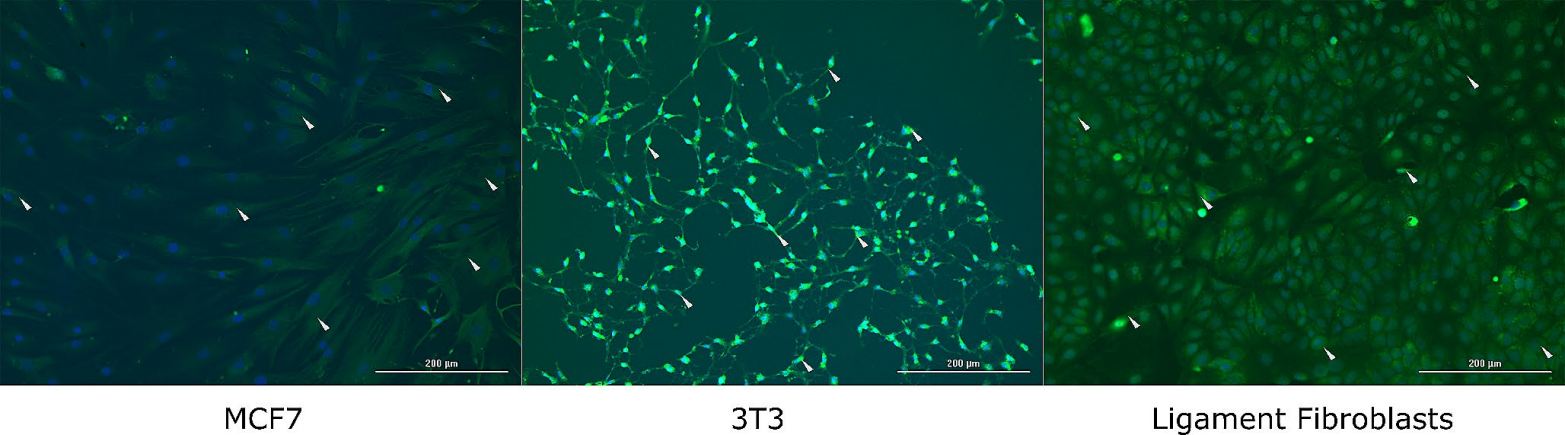
Nucleus and cytoskeleton differences between MCF-7, 3T3, and ligament fibroblasts using immunofluorescence analysis

The immunofluorescence analysis aims to confirm that the explanted cells are fibroblasts [17]. Images were obtained and analyzed based on morphological shape and staining intensity comparing three different fibroblast cell lines: breast cancer cells (MCF-7) as a negative control and mouse embryonic fibroblasts (3T3) as a positive control. Figure 2 shows MCF-7, 3T3, and ligament fibroblast cells tested through immunofluorescence analysis. MCF-7 cells exhibited a precise dome shape with darker spots within the nuclei, circumscribing the membrane. Additionally, staining with Anti-Vimentin was done to detect the typical cytoskeleton of fibroblast cells, and we observed less intense color on MCF-7 compared to ligament fibroblasts and 3T3 cells. We also noted a smaller size, with circumferential nuclei and less elongated than fibroblast cells. Morphological characteristics of human fibroblasts include a flat, oval-shaped nucleus and elongated fibril bundles (spindle-shaped) extending to the cell’s ends (Fig. 2). In the cytoskeleton, the Anti-Vimentin antibody activates a green color staining with Hoechst (33,342), allowing for the evaluation of nuclear morphology in the three cell cultures, revealing a light blue color.

### Cell viability

**Fig. 3:**
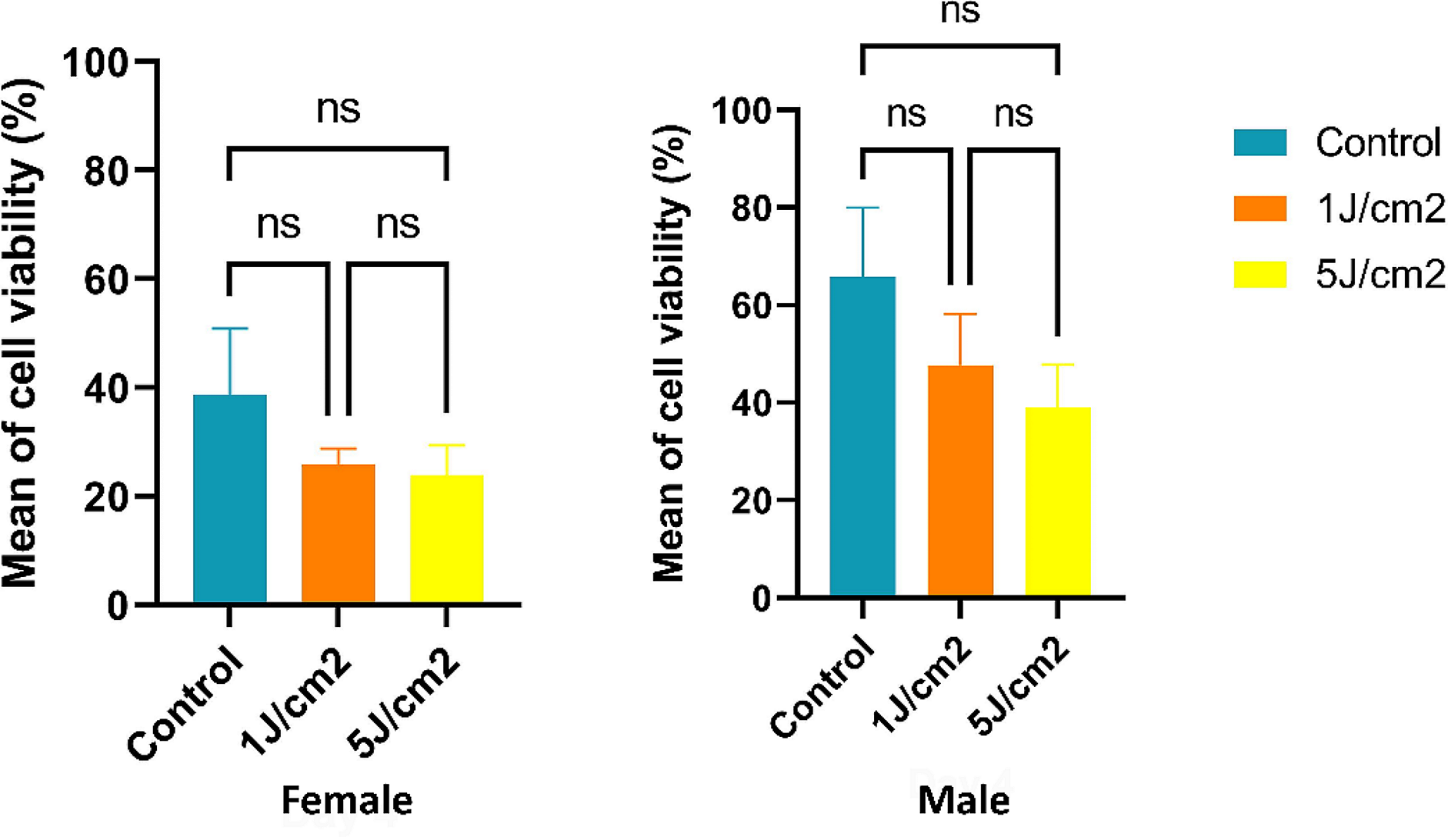
Cell viability decreased after LLLT treatment

The results for cell viability in the same proliferation assay were obtained, indicating the percentage of cell viability for each group. In female fibroblasts, on day 3, the control group experienced an increase in cell viability, showing a rise of 7.61% compared to the low dose and 5.73% compared to the high dose. After treatment, the control group exhibited a cell viability increase of 33.17% compared to the low dose and 38.21% to the high dose without statistical significance. On the other hand, in male fibroblasts, after treatment, the control group showed an increase in cell viability compared to the treatment groups. While the low dose decreased by 27.57%, the high dose decreased by 40.76% (Fig. 3).

### Cell proliferation

**Fig. 4:**
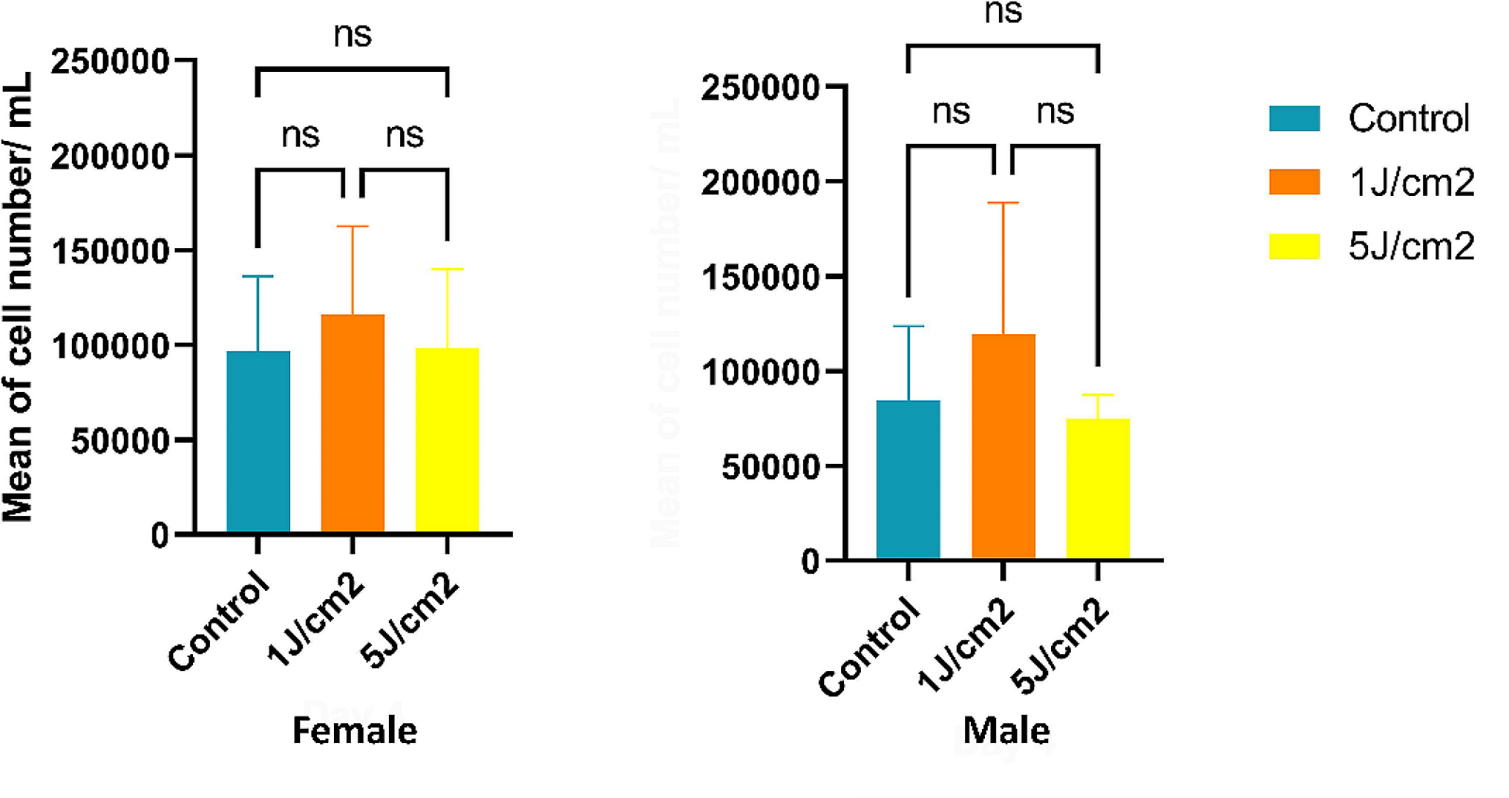
Cell proliferation increased after low LLLT treatment

Cell counting results, both through trypan blue staining and automated cell counting for the low dose, revealed an increase in the cell number of female and male fibroblasts after treatment. In female fibroblasts, on day 4, after LLLT treatment, the low dose exhibited a higher proliferation of 16.61% compared to the control group without statistical significance. In male fibroblasts, after treatment, the low dose showed an increase of 29.15% compared to the control group, although this value also did not reach statistical significance (Fig. 4). However, on day 3, treatments showed statistically significant increased cell number (*p* = 0.0164) compared to the control group. While the low dose increased by 43.87%, the high doses increased by 10% (see supplementary material S1- Cell proliferation).

### Collagen type I

**Fig. 5:**
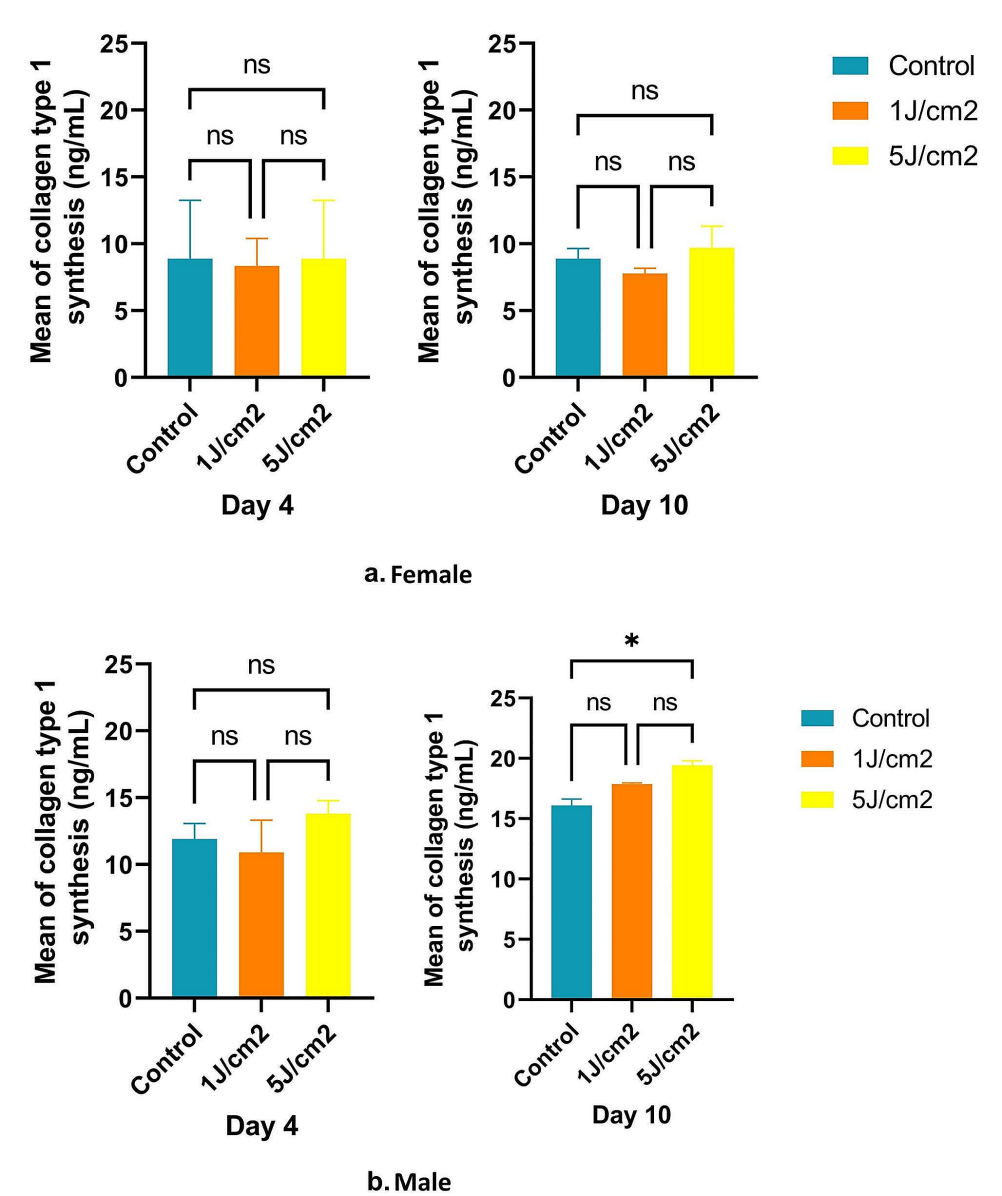
Collagen synthesis increased on day 10 after high LLLT treatment

The concentration of human COL1α1 in the cell culture medium was assessed at baseline, during, and after treatment. After treatment, no detectable differences were found between the control and the high dose in the culture medium of female samples (Fig. 5a). Although the high dose increased collagen synthesis by 5.92% compared to the control group, the difference of 0% was negligible and lacked statistical significance. However, by day 10, the high dose exhibited a significant increase of 8.49% compared to the control group and 19.75% compared to the low dose. These findings highlighted notable differences between groups, with a p-value of 0.0500, close to the statistical significance threshold.

Conversely, within male samples after treatment, the high dose exhibited a 13.73% increase in collagen synthesis compared to the control group and a 21.00% increase compared to the low dose, though without statistical significance (Fig. 5b). By day 10, the high dose significantly increased collagen synthesis by 17.14% compared to the control group and 8% compared to the low dose, with significant differences between groups (*p* = 0.0107).

## Discussion

Low-level laser therapy (LLLT) has accelerated wound healing, involving processes such as cell proliferation and type I collagen synthesis. However, significant variability in application parameters still exists [8–10]. This study addresses the lack of specific research on cellular changes induced by LLLT at low (1.0 J/cm^2^) and high dose (5.0 J/cm^2^), 850 nm, for three consecutively days in the fibroblasts obtained from the human anterior cruciate ligament. In this context, it is noteworthy that this study represents the first investigation conducted on human anterior cruciate ligament cells. It focuses on elucidating the biological impact of LLLT on fibroblasts in the intraarticular ligament of the knee, examining the effects of different doses on cell proliferation, viability, and type I collagen synthesis. The current lack of information underscores the importance of findings that may emerge from this study.

This study explored the potential of Low-Level Laser Therapy (LLLT) to promote fibroblast proliferation and facilitate collagen synthesis. The results indicated increased cell proliferation in the low-dose group from the third day onward, affecting both genders. This aligns with a study conducted on Human Gingival Fibroblasts (HGF3-PI 53) cells irradiated at 4.0 J/cm^2^ for three consecutive days, where an apparent increase in cell proliferation was evident at 24 h, progressing to greater significance at the 48 and 72-hour intervals post-irradiation [11]. Specifically, in female fibroblasts irradiated with a low dose on the fourth day evaluated, there was a 16.61% increase compared to the control group and a 15.06% increase compared to the high dose. However, it is crucial to note that these values did not reach statistical significance.

On the other hand, a significant increase was observed for male fibroblasts irradiated with a low dose on the third day, with a 43.87% increase compared to the control and a 23.50% increase compared to the high dose. Additionally, on the fourth day, there was a 29.15% increase for the low dose compared to the control and a 37.27% increase compared to the high dose, although the latter value did not reach statistical significance. These findings align with the literature, supporting the notion that the optimal effects of bio-stimulation in proliferation occur with lower irradiances, as fibroblasts respond more effectively to small doses of low-intensity laser [18, 19].

An optimal wavelength is crucial in laser therapy for monitoring cell cultures. Several studies have highlighted that the wavelength spectrum, ranging from red to near-infrared (660 nm to 905 nm), can expedite wound healing in soft tissues without causing harm [8–10, 19, 20]. Although various studies have demonstrated that irradiation at high laser doses can reduce cell viability, as evidenced in investigations using rabbit urethral scar fibroblasts exposed to laser therapy, where cells exposed to low-intensity doses did not undergo significant damage [10]. In contrast, another study on myoblasts exposed to laser demonstrated increased viability in cultures with 2% FBS after 48 and 72 h without showing a significant increase in ROS [20].

Our results support this conclusion, as no decrease in cell viability was observed in experiments with a wavelength of 850 nm (1.0 J/cm^2^ and 5.0 J/cm^2^) during the initial days of treatment for both male and female fibroblasts. However, on the fourth day, the last day evaluated, a decrease in viability was evident in the groups exposed to irradiation for both male and female fibroblasts. It is postulated that variations in cell viability may be attributed to additional phenomena, such as early senescence and even the induction of necrosis or accidental cell death due to temperatures generated by the laser. Additionally, in our primary cell cultures, the confirmation of early or late apoptosis and necrosis must be validated using other reliable methods to distinguish between populations of live and dead cells, such as flow cytometry. However, it is essential to note that the behavior of viability in these studies could be more precise, emphasizing the need for more evidence for a comprehensive understanding.

Low-level laser therapy (LLLT) in fibroblasts induces significant type I collagen gene expression. In a study utilizing the following parameters: 810 nm, 50 mW diode laser (energy: 4.0 J/cm^2^), on Human Gingival Fibroblasts (HGF3-PI 53), a substantial increase in type I collagen synthesis was revealed three days after laser irradiation. An almost five-fold increase in gene expression was observed through real-time PCR analysis compared to the control group during the same period [11].

Several researchers have proposed mechanisms of action linking laser application to increased collagen type I synthesis and other critical factors in wound healing. Low-level laser therapy (LLLT) has been observed to trigger a brief and modest increase in the production of reactive oxygen species, restoring redox balance by elevating antioxidant enzymes. This modification in the redox state activates intracellular signaling, intensifying redox-sensitive transcription factors and influencing enzymatic activation and cell cycle progression—crucial events in wound healing processes [21]. The low-level laser treatment process also constitutes a photochemical reaction inherently linked to photon absorption, associated with cytochrome C oxidase. LLLT promotes ATP synthesis and respiratory frequency improvement through this mechanism, thus fostering lesion repair [22].

However, compared with other studies in the literature, variations in methodologies and approaches are needed, such as different types of fibroblasts and diverse measurement techniques. Despite these differences, the results of this study revealed an increase in the concentration of type I collagen at high doses for ligament fibroblasts from both female and male samples on day 10 after treatment compared to the treated group at the low dose and control group. It is essential to highlight that collagen has a healing and assembly time frame [5–7], which is why a more significant increase in collagen concentration was observed on day 10.

Additionally, this study revealed that, on day 10, male cells exhibited a higher synthesis of type I collagen compared to female fibroblasts. A systematic review encompassing 242 observational or intervention studies with self-reported activity or knee-related outcomes after an ACL injury concluded that knee-related outcomes are inferior for females/girls compared to males/boys after an ACL injury [23]. This is why future research and studies with specific dosages for each gender should be conducted, as it is believed that the healing process may vary between them. However, using low-level laser therapy could be a promising alternative to ensure equity in the return to daily activities or sports after an ACL injury.

## Conclusion

In conclusion, our study has demonstrated that low-level laser therapy (LLLT) at 850 nm, administered every 24 h over three days at low doses (1.0 J/cm²), enhances ligament fibroblast proliferation and, at high doses (5.0 J/cm²), increases the concentration of human collagen type I. Thus, LLLT may improve the early stage of the anterior cruciate ligament healing process.

Future research endeavors could evaluate the impact of LLLT on alignment and collagen synthesis in human subjects through randomized clinical trials, acknowledging the potential influence of these devices on fibroblast regeneration in the context of ligament healing and sex differences.

## Electronic supplementary material

Below is the link to the electronic supplementary material.

Supplementary Material 1
