# Supplementary material for: In-vitro study on type I collagen synthesis in low-level laser therapy on the early ligament fibroblasts’ healing process
Source: Lasers Med Sci. 2024 Aug 29;39(1):225. doi: 10.1007/s10103-024-04151-7 (PMC11362177; doi:10.1007/s10103-024-04151-7)
Supplement: Supplementary file 1 — Supplementary Material 1 [file 10103_2024_4151_MOESM1_ESM.docx]

**In-vitro study on type I collagen synthesis in low-level laser therapy on the early ligament fibroblasts' healing process**

**Supplementary material S1**

**S1 - Cell viability**

Figs. S1 and S2 show ligament fibroblasts viability before (day 1), during (days 2 and 3), and after the Low-Level Laser Therapy (LLLT) treatment (day 4) for ligament fibroblasts from female and male ligament samples. After measuring the cell viability percentage on day 1, we immediately applied the LLLT. This means that on day 2, we verified the cell viability after 24 hours of the first LLLT dose, on day 3 of the second LLLT dose, and on day 4, the last LLLT irradiation.


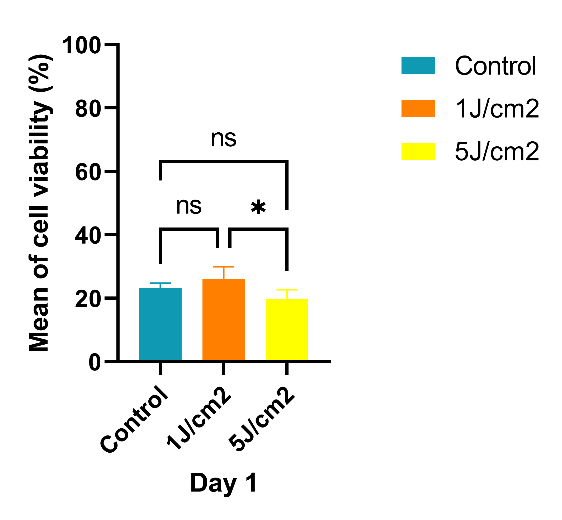

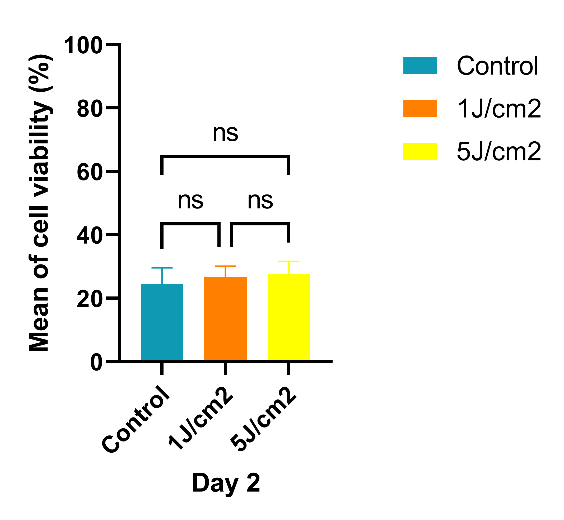

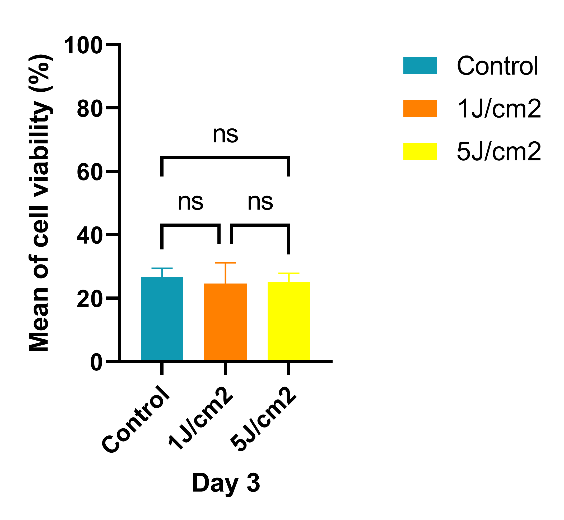

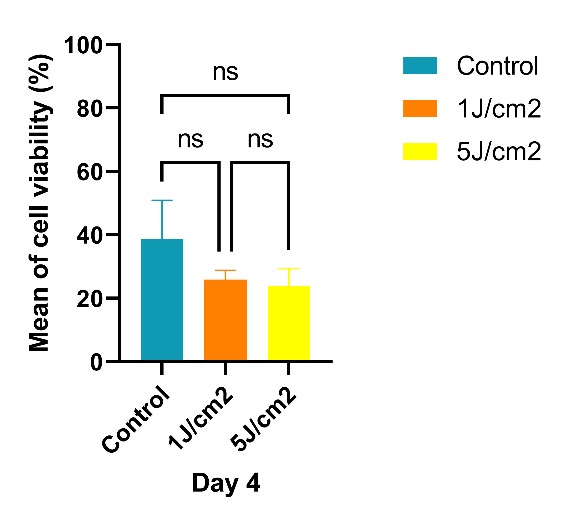


**Fig. S1** Ligament fibroblast viability before, during, and after the LLLT treatment for ligament fibroblasts from the female ligament sample


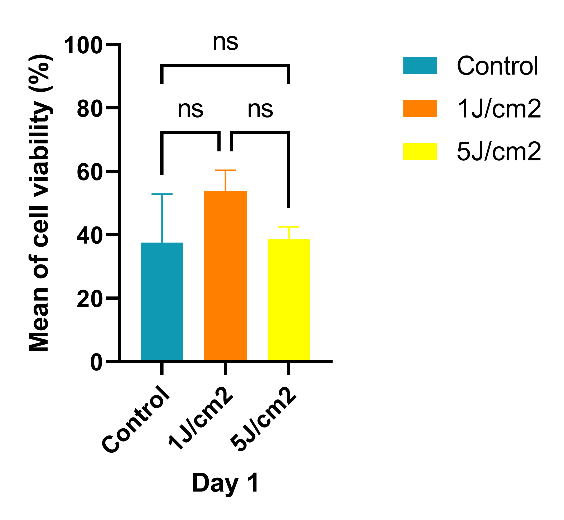

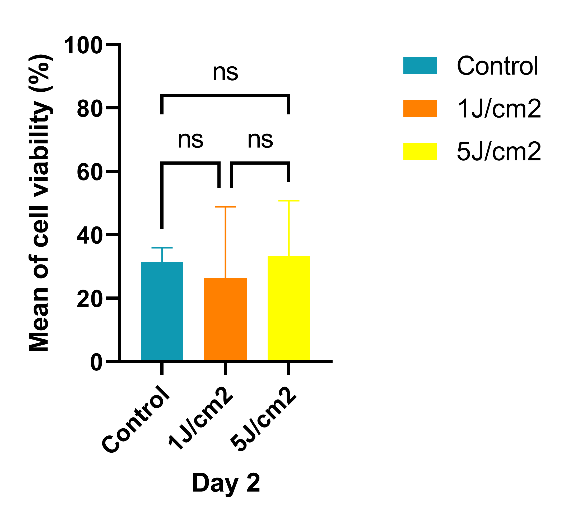

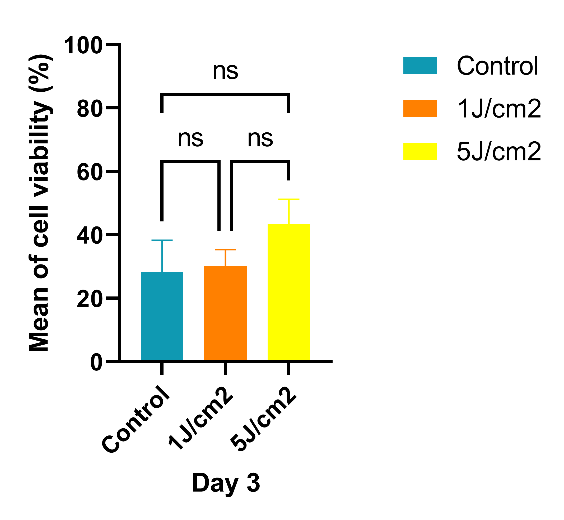

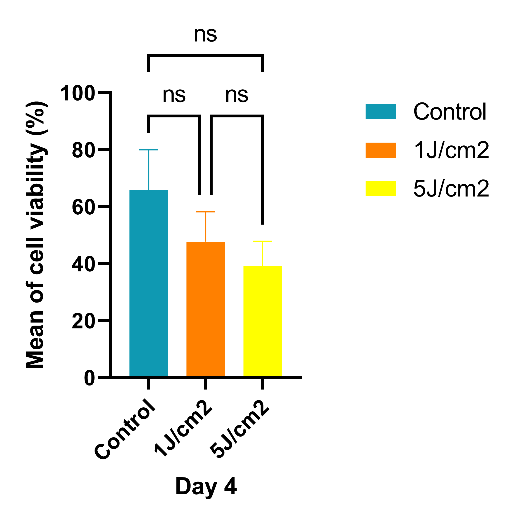


**Fig. S2** Ligament fibroblast viability before, during, and after the LLLT treatment for ligament fibroblasts from the male ligament sample

**S1 - Cell proliferation**

Figs. S3 and S4 show ligament fibroblasts proliferation behavior before (day 1), during (days 2 and 3), and after the Low-Level Laser Therapy (LLLT) treatment (day 4) for ligament fibroblasts from female and male ligament samples. After counting the cell number on day 1, we immediately applied the LLLT. This means that on day 2, we verified the cell number after 24 hours of the first LLLT dose, on day 3 of the second LLLT dose, and on day 4, the last LLLT irradiation.


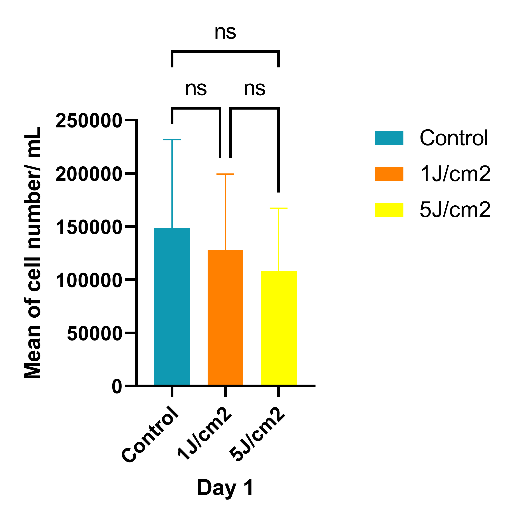

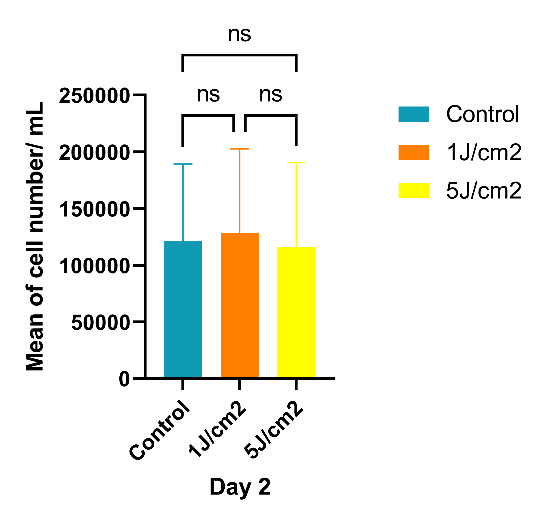

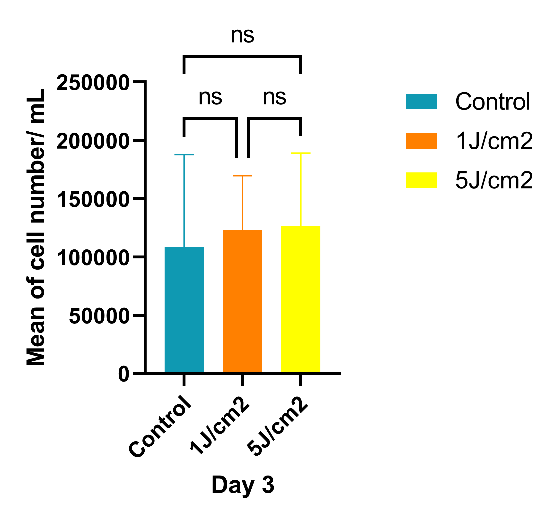

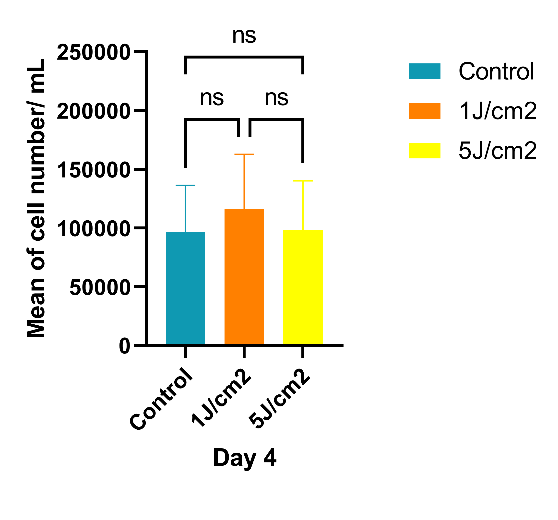


**Fig. S3** Ligament fibroblast proliferation behavior before, during, and after the LLLT treatment for ligament fibroblasts from the female ligament sample

**
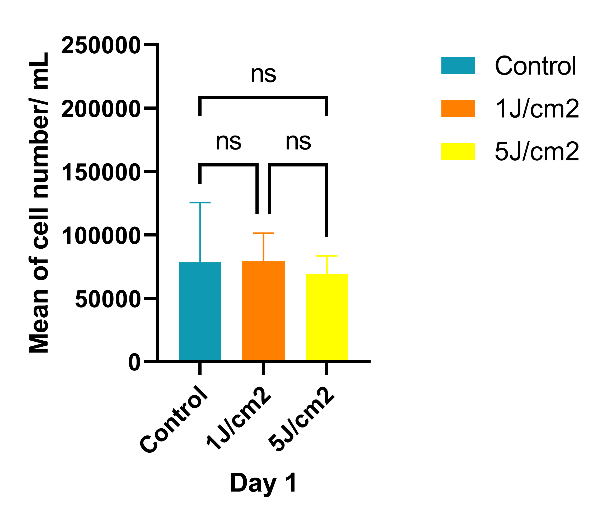

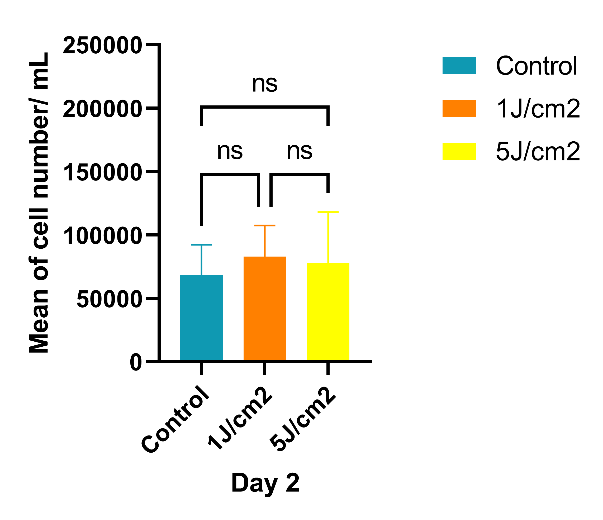

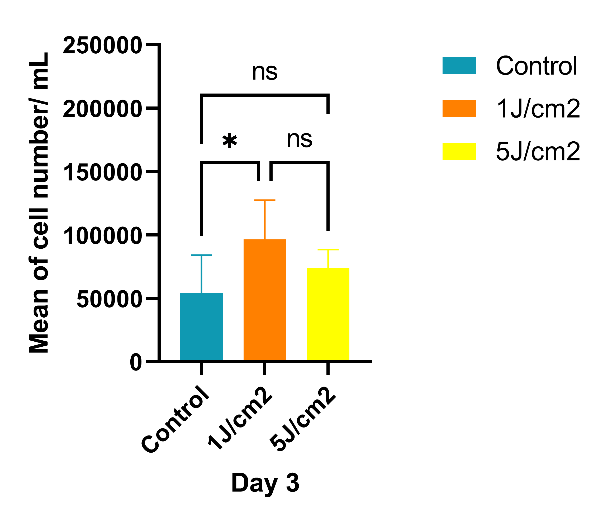

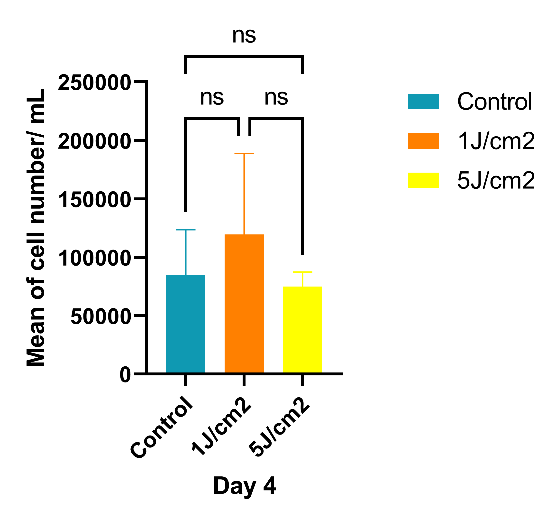
**

**Fig. S4** Ligament fibroblast proliferation behavior before, during, and after the LLLT treatment for ligament fibroblasts from the male ligament sample

**S1 - Collagen type I**

Figs. S5 and S6 show ligament fibroblasts synthesis of collagen type I before (day 1) and after the Low-Level Laser Therapy (LLLT) treatment (days 4 and 10) for ligament fibroblasts from female and male ligament samples. On day 1, cells were attached after 24h of seeding, and then we collected a medium sample and stored it for the following measurement using the immunoassay ELISA. On days 4 and 10, we did the same procedure.


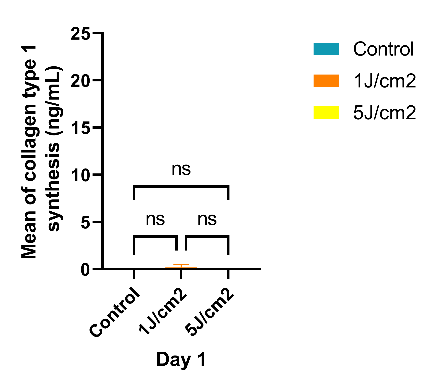

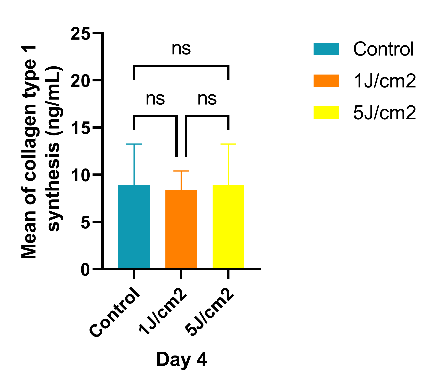

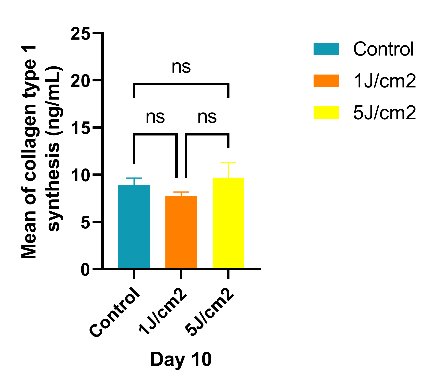


**Fig. S5** Ligament fibroblast synthesis of collagen type I before (day 1), and after the Low-Level Laser Therapy (LLLT) treatment (days 4 and 10) for ligament fibroblasts from the female ligament sample


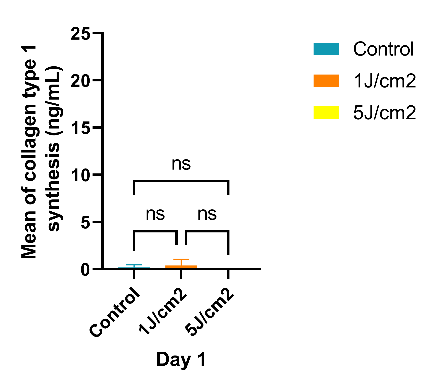

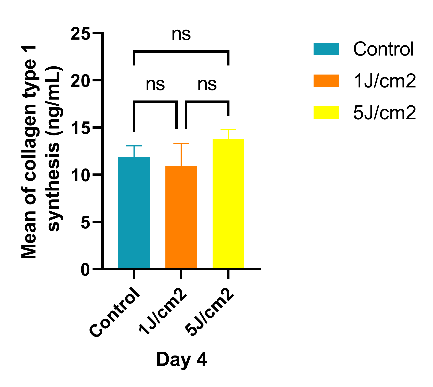

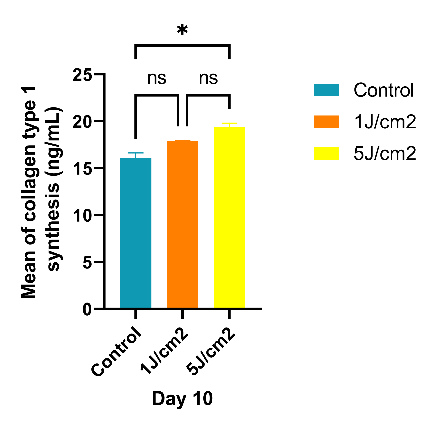


**Fig. S6** Ligament fibroblast synthesis of collagen type I before (day 1), and after the Low-Level Laser Therapy (LLLT) treatment (days 4 and 10) for ligament fibroblasts from the male ligament sample
